# Personal and environmental correlates of active travel and physical activity in a deprived urban population

**DOI:** 10.1186/1479-5868-5-43

**Published:** 2008-08-27

**Authors:** David Ogilvie, Richard Mitchell, Nanette Mutrie, Mark Petticrew, Stephen Platt

**Affiliations:** 1 Medical Research Council Social and Public Health Sciences Unit, Glasgow, UK; 2 Section of Public Health and Health Policy, University of Glasgow, Glasgow, UK; 3 Department of Sport, Culture and the Arts, University of Strathclyde, Glasgow, UK; 4 London School of Hygiene and Tropical Medicine, London, UK; 5 Research Unit in Health, Behaviour and Change, University of Edinburgh, Edinburgh, UK; 6 Medical Research Council Epidemiology Unit, Cambridge, UK

## Abstract

**Background:**

Environmental characteristics may be associated with patterns of physical activity in general or with particular types of physical activity such as active travel (walking or cycling for transport). However, most studies in this field have been conducted in North America and Australia, and hypotheses about putative correlates should be tested in a wider range of sociospatial contexts. We therefore examined the contribution of putative personal and environmental correlates of active travel and overall physical activity in deprived urban neighbourhoods in Glasgow, Scotland as part of the baseline for a longitudinal study of the effects of opening a new urban motorway (freeway).

**Methods:**

We conducted a postal survey of a random sample of residents (n = 1322), collecting data on socioeconomic status, perceptions of the local environment, travel behaviour, physical activity and general health and wellbeing using a new 14-item neighbourhood rating scale, a travel diary, the short form of the International Physical Activity Questionnaire (IPAQ) and the SF-8. We analysed the correlates of active travel and overall physical activity using multivariate logistic regression, first building models using personal (individual and household) explanatory variables and then adding environmental variables.

**Results:**

Active travel was associated with being younger, living in owner-occupied accommodation, not having to travel a long distance to work and not having access to a car, whereas overall physical activity was associated with living in social rented accommodation and not being overweight. After adjusting for personal characteristics, neither perceptions of the local environment nor the objective proximity of respondents' homes to motorway or major road infrastructure explained much of the variance in active travel or overall physical activity, although we did identify a significant positive association between active travel and perceived proximity to shops.

**Conclusion:**

Apart from access to local amenities, environmental characteristics may have limited influence on active travel in deprived urban populations characterised by a low level of car ownership, in which people may have less capacity for making discretionary travel choices than the populations studied in most published research on the environmental correlates of physical activity.

## Background

Until recently, research on correlates of physical activity was dominated by studies of individual demographic and psychosocial characteristics [1]. This reflected an emphasis on promoting sport, recreation or health-directed exercise using techniques to encourage individual behaviour change [2]. However, there is little evidence that such approaches are effective in increasing physical activity in the medium-to-long term [3]. If habitual patterns of behaviour are environmentally cued, sustained change is likely to require a supportive environment in which people can be active [4,5]. There is therefore increasing interest in the influence of the social and physical environment on physical activity.

With respect to the physical (natural or built) environment, a growing body of evidence suggests that certain environmental characteristics may be associated with patterns of physical activity in general or with particular types of physical activity such as walking or cycling as modes of transport [4-10]. Among the correlates most frequently identified in such reviews – some ascertained using 'objective' measures, and others in terms of people's perceptions – are the aesthetic quality of the surroundings, the presence of pavements (sidewalks), the convenience of facilities for being active, the availability of green space, access to amenities (destinations) within walking or cycling distance, safety from traffic and personal attack, and the lack of heavy traffic. Some of these local characteristics reflect higher-order aspects of urban design and spatial policy such as population density, connectivity and mixed land use [6,8]. Importantly, different characteristics may be associated with different types of physical activity; for example, Owen and colleagues found that the aesthetic quality of the surroundings was associated with walking for exercise or recreation and with walking in general, but not with walking for transport, whereas perceptions of traffic were associated with walking for transport and walking in general, but not with walking for exercise or recreation [5].

Despite the growing volume of published studies in this field, many authors remain circumspect in their interpretation of the available evidence. Giles-Corti and Donovan have described access to a supportive physical environment as a necessary, but insufficient, condition for an increase in physical activity in the population [11], while Handy found 'convincing' evidence of an association between physical activity and the built environment in general but 'less convincing' evidence as to which specific environmental characteristics were most strongly associated [7]. One limitation of the available evidence is that most research has been conducted in North America and Australia [9,12], and it is not clear whether associations observed in those countries are generalisable to other settings with different aggregate socioeconomic characteristics (e.g. wealth or access to private cars) or environmental characteristics (e.g. climate, patterns of land use, or availability of public transport). For example, North American researchers are often interested in the presence or absence of pavements (sidewalks), but it is unusual for streets in the United Kingdom (UK) not to have a pavement or footpath beside them. Hypotheses about putative environmental correlates of physical activity therefore need to be tested in a wider range of settings.

A more profound limitation of the available evidence is that identifying a relationship between, for example, urban form and walking for transport is not the same thing as showing that *changing *the built environment will lead to a change in behaviour [13]. Few researchers have taken up the opportunity (or challenge) presented by 'natural experiments' to investigate the effects of environmental interventions on physical activity [14]. We therefore established a longitudinal study to examine changes associated with the opening of a new urban section of the M74 motorway (freeway) currently under construction in Glasgow, Scotland. The rationale and design for this study have been described previously [15]. It is claimed that the new motorway, which will mostly pass through or close to densely-populated urban neighbourhoods, will contribute to the regeneration of a region which includes some of the most deprived and least healthy working-class communities in Europe [16]. It is also claimed that the new motorway will divert traffic from local streets, reduce traffic noise and bring new local employment opportunities, thereby improving characteristics of the local environment held to be associated with active travel. Others claim that the new motorway will encourage car use, degrade the aesthetic quality of the surroundings and reduce the safety and attractiveness of routes for pedestrians and cyclists across the line of the motorway – all changes which may be expected to discourage active travel [15]. The eventual aim of the M74 study will be to assess the effects of this major modification to the urban built environment and transport infrastructure on perceptions of the local environment and on population health and health-related behaviour, the primary outcome of interest being a change in the quantity of 'active travel' (walking and cycling for transport).

In this paper, we report findings from the cross-sectional (baseline) phase of the study which contribute evidence on the environmental correlates of physical activity in this comparatively deprived urban population. We focus on two specific hypotheses: first, that levels of active travel and overall physical activity vary with demographic and socioeconomic characteristics, but not necessarily in the same way; second, that these relationships may be partly explained by the perceived characteristics of the local environment in which people live and by their objectively-assessed proximity to motorway and major road infrastructure.

## Methods

### Delineation of study areas

**Figure 1:**
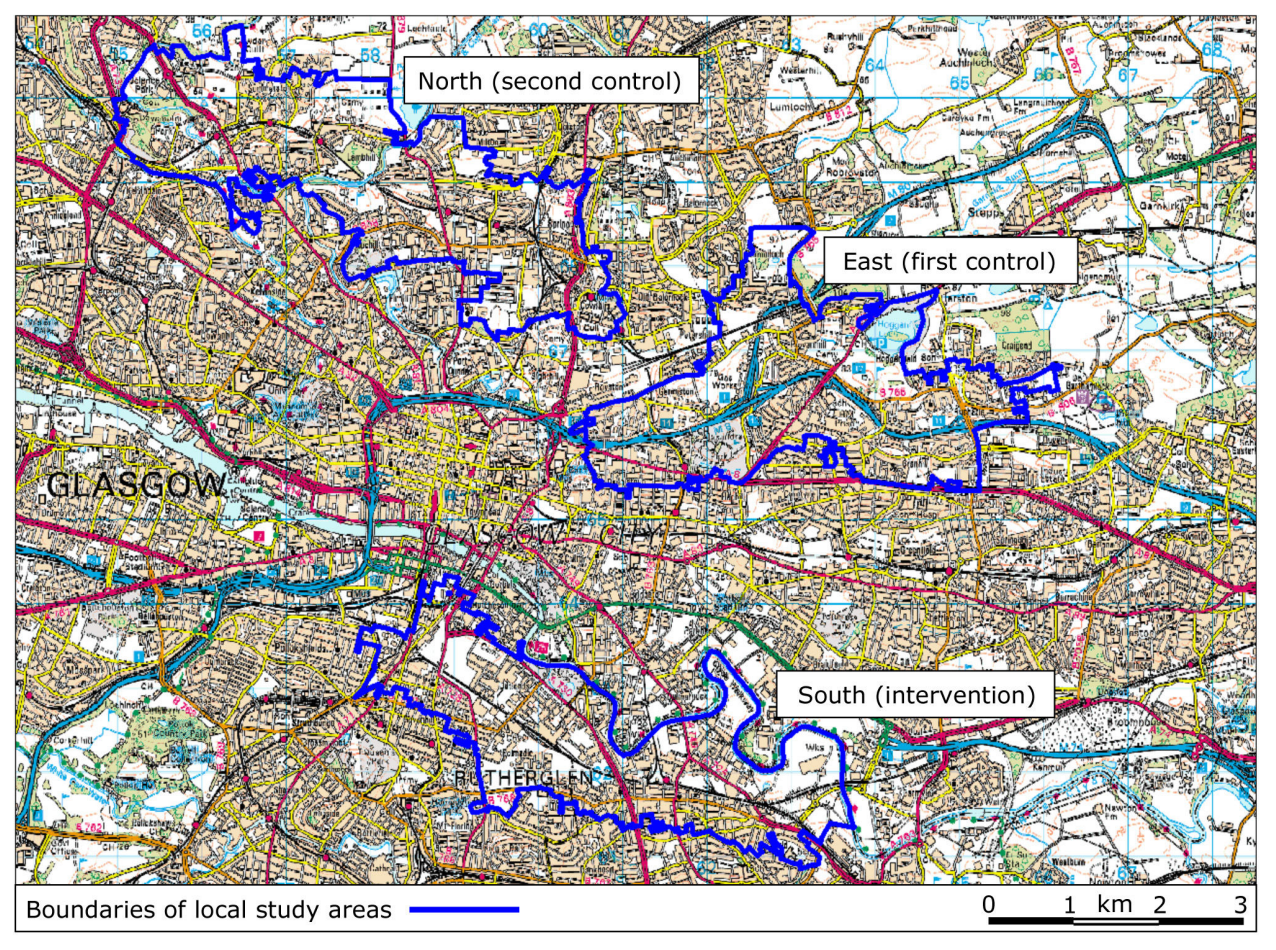
**Boundaries of local study areas defined in terms of census output areas**. Data and raster image ^© ^Crown Copyright/database right 2005. An Ordnance Survey/EDINA supplied service.

**Figure 2:**
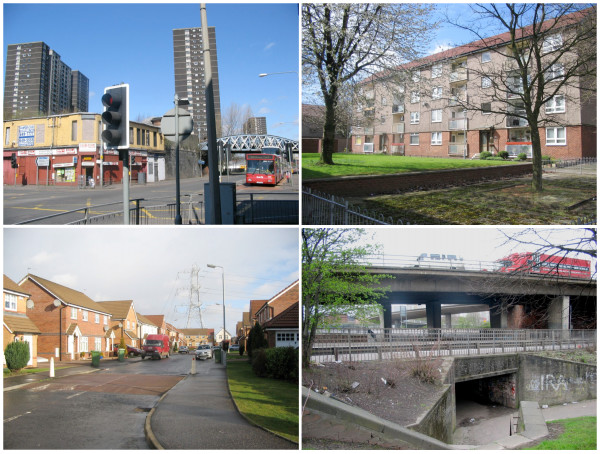
**Examples of scenes in and around the local study areas**. All images ^© ^David Ogilvie.

We used spatially referenced census and transport infrastructure data held and analysed in a geographical information system (GIS), combined with field visits, to delineate three study areas in Glasgow with similar aggregate socioeconomic characteristics and broadly similar topographical characteristics apart from their proximity to urban motorway infrastructure (Table 1, Figure 1). All three study areas extended from inner mixed-use districts close to the city centre to residential suburbs, contained major arterial roads other than motorways, and contained a mixture of housing stock including traditional high-density tenements, high-rise flats and new housing developments (Figure 2).

**Table 1:** Definitions of study areas

**Study area**	**Definition**
South	A set of census output areas (the smallest spatial unit for which aggregate census data are available) encroaching within 500 metres of the proposed route of the new M74 motorway
East	A set of census output areas encroaching within 500 metres of the routes of the existing M8 and M80 motorways
North	A set of census output areas not encroaching within 500 metres of the route of any existing or proposed motorway

### Sampling and survey administration

We used the Royal Mail Postcode Address File (PAF) (version 2005.3) to identify all residential addresses whose unit postcode (zip code) was within one of the study areas (total n = 35601) and drew a random sample of 3000 households from each area. Unit postcodes (e.g. G12 8RZ) are the smallest available unit of postal geography in the UK; residential unit postcodes cover about 15 addresses on average. We sent the survey to all households (total n = 9000) between 28 September and 4 October 2005 and resent the survey to all non-responding households between 26 and 31 October 2005. We alerted households to the survey by means of a postcard sent a few days in advance, used coloured paper for some of the survey materials, and posted survey packs in white envelopes printed with the university crest; these techniques have been shown in a meta-analysis to be associated with increased response rates to postal surveys [17]. We asked householders to ensure that the questionnaire was completed by a resident aged 16 or over; if more than one resident was eligible, we asked householders to select the person with the most recent birthday. Respondents who consented to follow-up were entered into a prize draw to win a £50 (€63; US$92) gift voucher. Responses received more than three months after the first mailing wave were disregarded in analysis.

### Data collection

The questionnaire included items on demographic and socioeconomic characteristics, health and wellbeing (including the the SF-8 scale), perceptions of the local environment, travel behaviour and the short form of the International Physical Activity Questionnaire (IPAQ) (Additional file 1). We developed a new 'neighbourhood scale' to assess perceptions of relevant characteristics of the local environment (aesthetics, green space, access to amenities, convenience of routes, traffic, road safety and personal safety). The development, principal components analysis and reliability of the items in this scale and the derivation and reliability of summary variables are reported in an accompanying paper [18].

### Data cleaning and derivation of variables

#### Demographic and socioeconomic characteristics

We excluded from analysis all respondents who failed to enter their age or sex. We then examined the distributions of all raw variables and carried out range and consistency checks to identify any anomalous values or variables with a high proportion of missing responses. As a consequence, we collapsed responses on distance to place of work or study, housing tenure, car access and working situation into fewer categories by merging categories with small numbers of responses; we also disregarded household composition and working situation of spouse or partner in analysis because of the large numbers of missing values for these variables.

#### Health and wellbeing

We calculated body mass index (BMI) by converting, where necessary, self-reported heights and weights from imperial to metric units and dividing the height in metres by the square of the weight in kilograms; we also categorised respondents into quintiles of BMI. We calculated physical (PCS-8) and mental (MCS-8) health summary scores from the SF-8 data and scaled these to population norms using the method and coefficients given in the SF-8 manual [19].

#### Objective environmental characteristics

We linked each record to the unit postcode of residence. We then constructed concentric buffers at 100-metre intervals up to 500 metres around the routes and access points of existing and planned motorways and around the network of other major (A- and B- class) roads, and assigned each respondent to a category of proximity to each type of road infrastructure (within 100 metres, 101–200 metres, etc.) based on the location of the centroid of their unit postcode.

#### Travel behaviour

For travel time analysis we included travel diaries which recorded no travel at all, but we disregarded travel data from respondents who had not been at home on the day of the travel diary, whose questionnaire had been misprinted such that the travel diary pages were unusable, who had recorded journeys without reporting valid quantitative data on the durations of those journeys, or whose completed travel diary appeared implausible. We also disregarded journeys whose purpose was not stated or was beyond the scope of the travel diary (Additional file 1, page 8). We summed the reported travel time for each mode of transport, calculated a total travel time by active modes (walking plus cycling) and by all modes combined, and calculated the proportion of total travel time contributed by each mode of transport.

#### Physical activity

We cleaned and analysed IPAQ data in accordance with the IPAQ scoring protocol . We therefore disregarded physical activity data from respondents who had reported more than 16 hours of physical activity per day or who had missing or internally inconsistent data on the frequency or duration of any of the three categories of physical activity (walking, moderate-intensity activity or vigorous activity). We also recoded reported durations of activity of less than ten minutes to zero, and of greater than 180 minutes to 180 minutes. We calculated the estimated total physical activity energy expenditure for each respondent (MET-min/week) and used a combination of frequency, duration and total energy expenditure to assign each respondent to a 'high', 'moderate' or 'low' category of overall physical activity in accordance with the prescribed IPAQ algorithm. The 'high' category corresponds to a sufficient level of physical activity to meet current public health recommendations for adults [20].

### Analysis

We considered it unlikely that the statistical assumptions required for linear regression could be met because the distributions of time spent walking and cycling and of estimated total physical activity energy expenditure were both strongly positively skewed and dominated by a large number of zero values which meant that the data were not amenable to log-transformation. We therefore modelled the correlates of active travel and physical activity using multivariate logistic regression. We defined 'active travel' as a binary condition achieved by any respondent who had reported at least 30 minutes of travel by walking, cycling or both in their travel diary, reflecting the current recommendation that adults should accumulate at least 30 minutes of moderate-intensity physical activity on most days of the week [20], and we defined 'physical activity' as a binary condition achieved by any respondent whose overall physical activity was categorised as 'high' using IPAQ. We then built separate multivariate models for active travel and physical activity following the method of Hosmer and Lemeshow [21], first including only 'personal' (individual or household) variables and then adding 'environmental' variables (Additional file 2).

## Results

### Response

We received 1345 completed questionnaires. After subtracting from the numerator 23 completed questionnaires with missing critical demographic data (age or sex), and after subtracting from the denominator 676 addresses from which survey packs were returned as undeliverable, this left 1322 valid responses to be entered into analysis – a response rate of 1322/(9000-676) = 15.9%.

### Characteristics of study participants

#### Demographic and socioeconomic characteristics

Respondents were aged between 16 and 89 years (median age 48 years). 804 (61%) were women. Only 136 (26%) of the men and 145 (18%) of the women reported having access to a bicycle. For those who usually travelled to a place of work or study, the median reported distance was 3.5 miles (about 5.5 kilometres). Other characteristics of study participants are summarised in Table 2.

**Table 2:** Socioeconomic characteristics of study participants

**Category**	**Frequency (%)**
**Working situation**	
Employed	616 (47.2)
Retired	333 (25.5)
Other*	357 (27.3)
Missing	16
**Financial situation**	
Find it a strain to get by from week to week	233 (17.9)
Have to be careful about money	680 (52.2)
Able to manage without much difficulty	299 (23.0)
Quite comfortably off	90 (6.9)
Missing	20
**Housing tenure**	
Owner-occupied	678 (51.6)
Social rented	543 (41.3)
Other^†^	93 (7.1)
Missing	8
**Cars or vans available to household**	
None	629 (48.4)
One	525 (40.4)
Two or more	146 (11.2)
Missing	22

n = 1322. * On a government training scheme, full-time student, unemployed, disabled, invalid or permanently sick, or caring for home and family or dependants. ^† ^Rented in the private sector, part-owned and part-rented, or other form of tenure.

#### Health and wellbeing

25% of respondents reported difficulty walking for a quarter of a mile, 39% reported a long-term health problem or disability, and 50% were overweight (median BMI 25.1 kg/m^2^). The median mental health summary score (MCS-8) was significantly lower (i.e. poorer) than the population norm (median 47.3, 95% CI 46.4 to 48.1); the median physical health summary score (PCS-8) was not significantly different from the population norm (median 50.9, 95% CI 49.6 to 51.7).

### Descriptive data on travel behaviour and physical activity

#### Travel behaviour

1099 travel diaries were suitable for travel time analysis. Men and women were equally likely to have returned usable travel time data, but respondents who were older, retired, or living in social rented accommodation or who did not have access to a car were less likely to have returned usable data. On average, respondents recorded about an hour's travel per day (mean 61.5 minutes, median 50.0 minutes), of which a minority was spent using active modes of transport (walking or cycling: mean 20.0 minutes, median 10.0 minutes) (Table 3). 304 respondents (28%) recorded at least 30 minutes of active travel, of whom 294 (97%) recorded at least 30 minutes of walking.

**Table 3:** Daily travel time by mode recorded in travel diaries

	**All respondents reporting valid travel time data**		
**Mode**			
	**Mean (sd)**	**Median (IQR) (range)**	**Proportion of total**
Car	24.4 (40.8)	0.0 (40.0) (0–510)	39.7%
Walking	19.2 (27.8)	10.0 (30.0) (0–205)	31.2%
Bus	14.6 (30.8)	0.0 (20.0) (0–210)	23.7%
Rail	1.8 (10.0)	0.0 (0.0) (0–165)	2.9%
Cycling	0.7 (7.3)	0.0 (0.0) (0–130)	1.1%
Motorcycle	0.1 (2.0)	0.0 (0.0) (0–50)	0.2%
Other	0.6 (9.4)	0.0 (0.0) (0–240)	1.0%
Active modes*	20.0 (28.5)	10.0 (30.0) (0–205)	32.4%
All modes combined	61.5 (53.2)	50.0 (63.0) (0–510)	100.0%

n = 1099. sd: standard deviation. IQR: interquartile range. * Walking and cycling combined.

#### Physical activity

833 respondents returned complete physical activity data suitable for analysis. Women and respondents who were older, retired, or living in social rented accommodation or who did not have access to a car were less likely to have returned usable data. Respondents reported a mean of 318 minutes' walking per week and a mean estimated total physical activity energy expenditure of 3000 MET-minutes per week (Table 4). Only 316 respondents (38%) were categorised as having achieved a 'high' (i.e. sufficient) level of physical activity.

**Table 4:** Average time spent walking and total physical activity

**Summary measure**	**Mean (standard deviation)**	**Median (interquartile range) (range)**
Walking (min/week)	318.4 (366.1)	180.0 (375.0) (0–1260)
Total activity (MET-min/week)	3000.1 (3323.1)	1935.0 (3645.0) (0–18438)

n = 833

### Correlates of active travel

**Figure 3:**
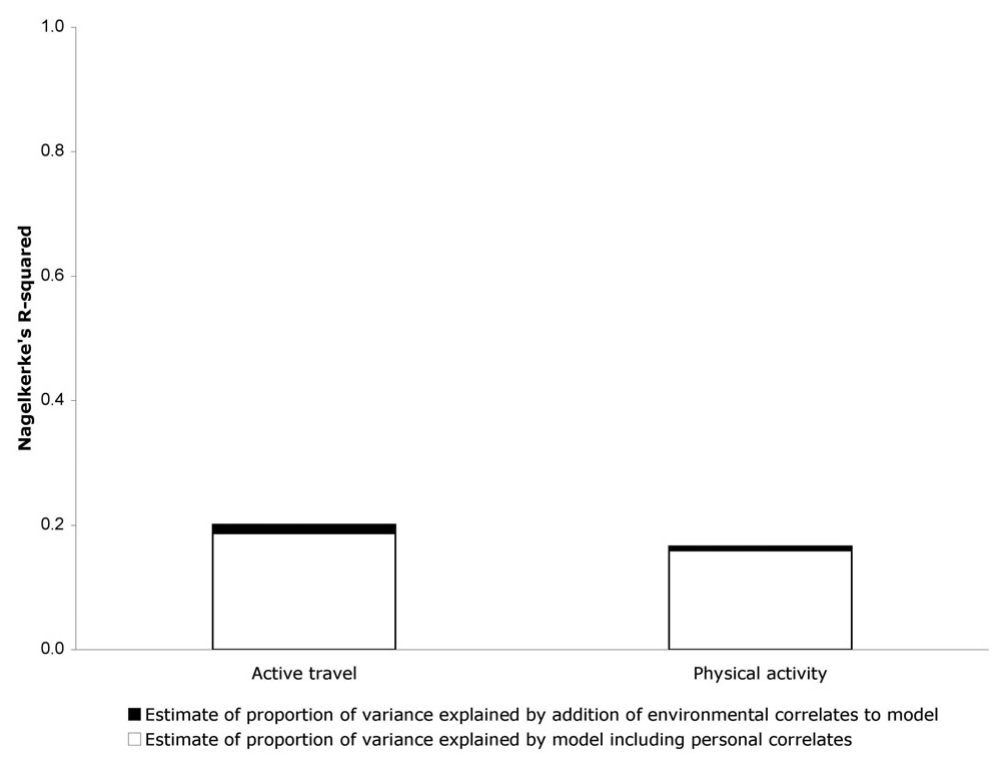
Estimated proportions of variance in active travel and physical activity explained by personal and environmental characteristics.

Active travel was significantly associated with being younger, living in owner-occupied accommodation, not having to travel more than four miles to work, having access to a bicycle, not having access to a car, and the absence of any difficulty walking. The final best model of the 'personal' correlates of active travel provided satisfactory goodness-of-fit (Hosmer and Lemeshow test: χ^2 ^= 13.04, df = 8; P = 0.11) and explained nearly one-fifth of the total variance in active travel (Nagelkerke's R^2 ^= 18.7%) (Table 5). Adding 'environmental' variables to the model showed an additional significant positive association between active travel and perceived proximity to shops, and an additional significant negative association between active travel and perceived road safety for cyclists. The final best model of the personal and environmental correlates of active travel also provided satisfactory goodness-of-fit (Hosmer and Lemeshow test: χ^2 ^= 10.61, df = 8; P = 0.23) and explained slightly more of the total variance in active travel than did the personal model alone (Nagelkerke's R^2 ^= 20.1%) (Figure 3).

**Table 5:** Multivariate logistic regression models of correlates of active travel

	**Model including personal correlates**		**Model including personal and environmental correlates**	
**Variable**	**OR (95% CI)**	**P**	**OR (95% CI)**	**P**
**Age**	0.98 (0.97, 0.99)	<0.001	0.98 (0.97, 0.99)	0.001
**Housing tenure (reference: social renter)**				
Owner-occupier	1.79 (1.19, 2.69)	0.005	1.70 (1.13, 2.58)	0.012
Other	1.64 (0.83, 3.24)	0.159	1.62 (0.81, 3.23)	0.17
**Distance to place of work or study (reference: four miles or more)**				
Less than four miles	1.76 (1.16, 2.68)	0.008	1.81 (1.18, 2.76)	0.006
Not applicable*	2.12 (1.27, 3.54)	0.004	2.15 (1.28, 3.61)	0.004
**Access to bicycle (reference: no)**				
Yes	1.59 (1.07, 2.35)	0.021	1.57 (1.06, 2.33)	0.025
**Composite variable (reference: access to car and difficulty walking)**				
Car, no difficulty	4.21 (1.43, 12,43)	0.009	3.77 (1.27, 11.23)	0.017
No car, difficulty	4.65 (1.48, 14.54)	0.008	4.42 (1.40, 13.92)	0.011
No car, no difficulty	14.06 (4.84, 40.80)	<0.001	12.88 (4.41, 37.67)	<0.001
**Individual items in neighbourhood scale**				
Proximity to shops			1.20 (1.02, 1.41)	0.031
Road safety for cyclists			0.83 (0.70, 0.98)	0.024
**Day of travel diary (reference: weekend)**				
Weekday	1.96 (1.32, 3.00)	0.001	1.91 (1.26, 2.89)	0.002

n = 831. * Does not work or study or usually works at home or from home. OR: Exponent of estimated regression coefficient, i.e. estimated odds ratio. 95% CI: 95% confidence interval for estimated odds ratio.

In order to aid interpretation, we also partitioned the dataset into two strata ('No car available' and 'Car available') and refitted the final model separately to each stratum of the dataset (Table 6). This showed that the subset of respondents with no access to a car accounted for the significant overall relationship between active travel and access to a bicycle, whereas those with access to a car accounted for the significant overall relationships with distance to place of work or study and perceptions of the local environment. The relationship with difficulty walking was also stronger in this group than in those without access to a car.

**Table 6:** Multivariate logistic regression model of personal and environmental correlates of active travel stratified by availability of a car

	**No car available**		**Car available**	
**Variable**	**OR (95% CI)**	**P**	**OR (95% CI)**	**P**
**Age**	0.98 (0.97, 1.00)	0.029	0.97 (0.95, 0.99)	0.008
**Housing tenure (reference: social renter)**				
Owner-occupier	1.57 (0.94, 2.65)	0.087	1.77 (0.86, 3.64)	0.12
Other	1.49 (0.61, 3.62)	0.38	1.64 (0.51, 5.30)	0.41
**Distance to place of work or study (reference: four miles or more)**				
Less than four miles	1.20 (0.57, 2.53)	0.63	1.96 (1.14, 3.37)	0.015
Not applicable*	1.00 (0.48, 2.11)	1.00	4.84 (2.20, 10.66)	<0.001
**Access to bicycle (reference: no)**				
Yes	2.17 (1.10, 4.29)	0.026	1.43 (0.86, 2.38)	0.17
**Difficulty walking (reference: yes)**				
No	2.49 (1.35, 4.57)	0.003	5.60 (1.74, 17.98)	0.004
**Individual items in neighbourhood scale**				
Proximity to shops	1.10 (0.88, 1.37)	0.39	1.34 (1.03, 1.74)	0.032
Road safety for cyclists	0.89 (0.71, 1.12)	0.31	0.77 (0.06, 0.99)	0.038
**Day of travel diary (reference: weekend)**				
Weekday	1.22 (0.71, 2.11)	0.47	3.32 (1.62, 6.82)	0.001

n = 831. * Does not work or study or usually works at home or from home. OR: Exponent of estimated regression coefficient, i.e. estimated odds ratio. 95% CI: 95% confidence interval for estimated odds ratio.

### Correlates of physical activity

Physical activity was significantly associated with living in social-rented accommodation, not being overweight, and the absence of any difficulty walking. The final best model of the 'personal' correlates of physical activity provided satisfactory goodness-of-fit (Hosmer and Lemeshow test: χ^2 ^= 3.89, df = 7; P = 0.89) and explained about one-sixth of the total variance in physical activity (Nagelkerke's R^2 ^= 15.9%) (Table 7). Adding 'environmental' variables to the model showed an additional significant negative association between physical activity and perception of traffic volume (i.e. respondents who perceived there to be a higher volume of traffic were more likely to report physical activity). The final best model of the personal and environmental correlates of physical activity also provided satisfactory goodness-of-fit (Hosmer and Lemeshow test: χ^2 ^= 3.86, df = 8; P = 0.87) and explained slightly more of the total variance in physical activity than did the personal model alone (Nagelkerke's 16.6%) (Figure 3).

**Table 7:** Multivariate logistic regression models of correlates of physical activity

	**Model including personal correlates**		**Model including personal and environmental correlates**	
**Variable**	**OR (95% CI)**	**P**	**OR (95% CI)**	**P**
**Housing tenure (reference: social renter)**				
Owner-occupier	0.67 (0.46, 0.96)	0.028	0.66 (0.46, 0.95)	0.026
Other	1.41 (0.72, 2.79)	0.32	1.45 (0.73, 2.87)	0.29
**Composite variable (reference: BMI≥25 and difficulty walking)**				
BMI<25, no difficulty	5.49 (2.97, 10.16)	<0.001	5.55 (3.00, 10.28)	<0.001
BMI<25, difficulty	0.32 (0.10, 1.01)	0.053	0.31 (0.10, 0.98)	0.047
BMI≥25, no difficulty	3.93 (2.11, 7.32)	<0.001	3.92 (2.10, 7.31)	<0.001
**Individual items in neighbourhood scale**				
Traffic volume			0.84 (0.70, 1.00)	0.050
**Day of travel diary (reference: weekend)**				
Weekday	0.64 (0.44, 0.93)	0.019	0.62 (0.43, 0.91)	0.015

n = 684. OR: Exponent of estimated regression coefficient, i.e. estimated odds ratio. 95% CI: 95% confidence interval for estimated odds ratio.

## Discussion

### Principal findings

In this deprived urban population, the likelihood of reporting active travel was associated with being younger, living in owner-occupied accommodation, not having to travel a long distance to work and not having access to a car, whereas overall physical activity was associated with living in social-rented accommodation and not being overweight. After adjusting for individual and household characteristics, neither perceptions of the local environment nor the objective proximity of respondents' homes to motorway or major road infrastructure appeared to explain much of the variance in active travel or overall physical activity, although we did find a significant positive association between active travel and perceived proximity to shops.

### Representativeness and completeness of survey data

Our difficulty in obtaining a representative sample of the resident population is not unique to our study. Although our final response rate was low, it was almost identical to that achieved in a recent population-based intervention study elsewhere in Glasgow [22]. Some of the challenges of recruiting research participants in areas of deprivation have been described elsewhere [23]; these are superimposed on a downward trend in participation in even the best-resourced national population surveys [24] and an upward (and socially biased) trend in opt-outs from the main alternative sampling frame, the edited electoral register [25]. Although our achieved sample contained a higher proportion of respondents from owner-occupied and car-owning households than predicted from 2001 census data for the same census output areas, these differences may be partly accounted for by an upward background trend in owner occupation and car access between 2001 and 2005. Our achieved sample is still clearly disadvantaged overall, in terms of socioeconomic and health status, compared with the country as a whole. It also contains sufficient heterogeneity to enable us to examine, in time, how the effects of the intervention are distributed between socioeconomic groups. We therefore consider our achieved sample fit for purpose.

We had to disregard a substantial proportion of cases in analysis because respondents had returned unusable travel time data or had returned physical activity data that were incomplete, internally inconsistent or included a 'Don't know' response and were therefore unacceptable according to the IPAQ scoring protocol. Most published studies using the same, short form of IPAQ have either not reported the distribution of the continuous summary measures or have not reported data for the UK separately from those for other countries where higher levels of physical activity are reported. Despite the high proportion of missing physical activity data in our dataset, however, the aggregate continuous data we obtained were broadly comparable to those reported in Rütten and colleagues' study of a random sample of UK adults [26]. We could have included more cases in physical activity analysis by, for example, imputing missing values, but the results would not have been comparable with others' owing to the substantial deviations from the scoring protocol which would have been required. The frequency of unusable responses was not reported in the international multi-centre study which originally established the validity and reliability of IPAQ [27]. It is possible that offering a 'Don't know' option in the self-completed IPAQ questionnaire encourages respondents to select this rather than to enter what may be a reasonably precise estimate of the actual time spent in physical activity; the respondent has no way of knowing that a single 'Don't know' response will result in all of their physical activity data being disregarded in analysis. This should be considered in any future revision of the IPAQ questionnaire and scoring protocol.

### Contribution of active travel to overall physical activity

The explanatory variables that were significantly associated with active travel but not with physical activity (distance to place of work or study, access to a bicycle, access to a car, perceived proximity to shops, and perceived road safety for cyclists) all have an obvious intuitive relationship with the use of walking or cycling as modes of transport. That they were not significantly associated with overall physical activity suggests either that active travel contributes only a minority of respondents' overall physical activity or that other factors not measured in this study are more important correlates of overall physical activity than those which determine active travel. A crude comparision of the quantity of active travel reported in the one-day travel diaries with the quantities of physical activity reported using IPAQ suggests that on average, active travel may indeed make only a small (~15%) contribution to overall physical activity in this study population. However, the real contribution may be substantially greater than this if, as has been shown previously, respondents tend to over-report their physical activity using IPAQ [28]. There can be little doubt that active travel makes a substantial contribution to the total quantity of *walking *reported in this study population. Irrespective of the true contribution of active travel to overall physical activity, however, it remains likely that other unmeasured personal and social factors beyond the scope of this study may be more important correlates of overall physical activity.

### Socio-spatial patterning of active travel and overall physical activity

Respondents living in owner-occupied households were more likely to report active travel than those living in social-rented accommodation, but less likely to report sufficient overall physical activity. Since neither working situation nor perceived financial situation emerged as significantly associated with active travel or overall physical activity, housing tenure and car access are the remaining explanatory variables in this dataset which can be interpreted as markers of socioeconomic status. Although having access to a car clearly reflects the possession of a material asset, it has been argued that this is a less direct marker of socioeconomic status than some other markers because, in Scotland at least, access to a car is a more-or-less essential requirement for living in many rural areas, whereas it is possible to live in a dense urban settlement such as Glasgow without using a car. In the final models in this study, therefore, housing tenure may be regarded as the primary marker of socioeconomic status. The findings consequently suggest conflicting socioeconomic gradients in prevalence: more advantaged respondents were more likely to report active travel, but more disadvantaged respondents were more likely to report sufficient overall physical activity. The higher prevalence of sufficient overall physical activity among the more disadvantaged despite their lower propensity for active travel is likely to reflect higher quantities of physical activity in other domains, particularly occupational and domestic activities, since leisure-time physical activity tends to be higher among more advantaged groups [29].

### Environmental characteristics: paradoxical, unmeasured, or irrelevant?

The two environmental variables that emerged as significantly associated with active travel, particularly among those without access to a car, were perceived proximity to shops and perceived road safety for cyclists. The positive association with perceived proximity to shops suggests that for active travel to be undertaken in this population, it may be more important that people live close to the amenities they need than that they live in an environment with more favourable subjective or discretionary considerations such as attractiveness or noise. This would be consistent with an understanding that walking as a mode of transport is primarily a way of undertaking journeys which have to be made anyway, as opposed to more discretionary (recreational) forms of walking which may be more susceptible to the influence of less-structural characteristics.

Although the negative association with perceived road safety for cyclists appears counter-intuitive, similar 'paradoxical inverse relationships' have been reported elsewhere, for example by Titze and colleagues in a study of the correlates of cycling among students [30] and by Humpel and colleagues in a study of correlates of walking for pleasure [31]. Titze and colleagues suggest that respondents who cycle regularly are more likely to be aware of, and report, the danger posed by traffic than non-cyclists or infrequent cyclists. A similar phenomenon could explain the negative association between physical activity and perception of traffic volume.

Overall, the influence of the putative environmental characteristics examined in this study on active travel and physical activity appeared small compared with that of the personal characteristics found to be significant, and including environmental characteristics in the models did not substantially modify the influence of personal characteristics.

On the one hand, this could reflect an artefact of the research methods (a false negative error), which could have arisen in various ways. In particular, the 'wrong' environmental exposure may have been measured, in that the environmental characteristics examined were those of the immediate surroundings of respondents' homes, whereas the propensity to choose active modes of transport may be more strongly influenced by the characteristics of the environment elsewhere on their routes [30], for example the perceived danger of cycling in the city centre – an association which may be absent, or at least diluted, when the 'exposure' examined is limited to the residential environment. It could also be argued that the apparently weak influence of environmental characteristics in this study reflects a reliance on respondents' perceptions which have not been objectively verified and may therefore be a weak proxy for the 'true' objectively-measured characteristics of their surroundings. However, as recent reviews have pointed out, the current weight of evidence for objective environmental correlates of walking is no greater than that for subjective environmental correlates [5] and it is entirely plausible that people's perceptions of their environment may be at least as important as their objective conditions in influencing their behaviour [6].

On the other hand, we may have demonstrated a real absence of any major association. Although at first sight this appears at odds with the growing body of review-level evidence for environmental correlates of physical activity, Wendel-Vos and colleagues noted that of all the environmental factors examined in all the studies included in their review, analysis showed a 'null association' in 76% of cases [9], and our finding that personal factors account for a much larger proportion of the variance in active travel or physical activity than is accounted for by environmental factors is consistent with those of some other European studies [32,33]. In the particular context of this study, residents may simply have adapted to adverse conditions in their local environment in the ways identified by Hedges in a qualitative study of people living close to new roads built in the UK in the 1970s [34] – particularly by attitudinal adaptation, which Hedges characterises as developing an attitude that it is futile to resist. One can imagine that in the most deprived areas of Glasgow, people may have become resigned to the nature of their surroundings, seeing them as inevitable and not amenable to change either through environmental improvement or through their moving to another area.

## Conclusion

After demographic and socioeconomic characteristics were taken into account, neither perceptions of the local environment nor objective proximity to major road infrastructure appeared to explain much of the variance in active travel or overall physical activity in this study. Our study population may be both objectively constrained by their socioeconomic circumstances (including comparatively limited access to private cars) and adapted to living in conditions which others would consider to pose a barrier to active travel. Under these circumstances, environmental characteristics which have been found to influence discretionary active travel in studies in other, more affluent populations may simply be irrelevant in a population which is more captive in its travel choices. Environmental correlates of active travel should not be assumed to be generalisable between populations; researchers should continue to test hypotheses about putative environmental correlates in different settings, and policymakers should recognise that the effects of interventions to change the environment are likely to vary between populations and between socioeconomic groups within populations.

## Competing interests

This paper is based on material contained in the first author's PhD thesis.

## Authors' contributions

DO had the original idea for the study, designed the study and the survey materials, applied for ethical approval, cleaned and coded the survey data, carried out all the geographical and statistical analyses and wrote the paper. MP was DO's PhD supervisor. RM, NM, MP and SP constituted the steering group for the study, contributed to and advised on the design of the study and the interpretation of the emerging findings, and contributed to the critical revision of the paper. All authors read and approved the final manuscript.

## Supplementary Material

Additional file 1 Survey questionnaire Click here for file

Additional file 2 Further details of multivariate logistic regression modelling Click here for file
