# Supplementary material for: Personal and environmental correlates of active travel and physical activity in a deprived urban population
Source: Int J Behav Nutr Phys Act. 2008 Aug 27;5:43. doi: 10.1186/1479-5868-5-43 (PMC2538543; doi:10.1186/1479-5868-5-43)
Supplement: Additional file 2 — Further details of multivariate logistic regression modelling [file 1479-5868-5-43-S2.pdf]

Additional file 2

Ogilvie et al, Int J Behav Nutr Phys Act 2008

Further details of multivariate logistic regression modelling

Methods

We began by entering each putative 'personal' explanatory variable (reflecting the demographic or socioeconomic characteristics of the individual or their household, or measures of their individual health or wellbeing) into a univariate logistic regression model to estimate the odds ratio (OR) for active travel, and the associated P-value, for each category of the explanatory variable compared with its reference category. Adopting the approach recommended by Hosmer and Lemeshow [21], we then selected variables for entry into a preliminary multivariate 'personal' model if they met the generous criterion of $P<0.25$ in univariate analysis; we also included the sex of respondents because the associations between walking and environmental characteristics have been shown to vary between the sexes in some studies [5]. However, we noted that the 'health and wellbeing' variables other than BMI were strongly associated with each other (i.e. collinear) and therefore decided to enter only one such variable, difficulty walking, into the model on the grounds that it was the variable with the largest odds ratio in univariate analysis (3.64), confirming the intuitive expectation that it would be more strongly associated with active travel than the other 'health and wellbeing' variables. We entered the selected 'personal' variables into a multivariate model along with two variables (whether they had recorded their travel on a weekday or at the weekend, and the study area of residence) to control for possible effects of sampling variation. We removed the variables which no longer appeared to be significant after adjustment for the other variables in this model and refitted the model. We then considered the possibility of interaction between the two variables reflecting access to particular modes of transport (bicycles and cars) and between each of those variables and age, distance to place of work or study, and difficulty walking, and added interaction terms for each pairwise combination of these variables to the model one at a time.

Having identified the optimal 'personal' model including interaction terms, we then proceeded to examine the contribution of 'environmental' variables to this model. We considered separately the influence of 'objective' characteristics (proximity to motorway and other major road infrastructure) and 'subjective' characteristics (perceptions of the local environment), and within the 'subjective' domain we examined the independent contribution of each item on the neighbourhood scale as well as a variety of summary measures. By definition, there was a degree of overlap or collinearity between the various summary measures, which is why it would not have been appropriate to enter them all into a model simultaneously. Instead, we entered both individual and summary measures separately as an exploratory exercise to see which, if any, of these would contribute significantly to an overall model and selected the most promising to be retained. We then continued to remove variables and refit the model until we had reached the most parsimonious, stable and well-fitting model possible. We checked the goodness-of-fit of alternative models using the Hosmer and Lemeshow test and compared the proportion of the variance in active travel explained by alternative models using Nagelkerke's R^2.

We used an analogous procedure to model the correlates of physical activity.

Results

Correlates of active travel

Multivariate model of personal correlates

In univariate analysis, seven 'personal' variables met the criterion (P<0.25) to be considered in multivariate analysis: age, housing tenure, working situation, distance to place of work or study, access to a bicycle, number of cars available, and quintile of BMI. When we entered these variables into a multivariate model along with sex, difficulty walking and the two control variables (day of travel diary and study area of residence), four variables (sex, working situation, quintile of BMI, and study area of residence) no longer appeared to be significant. We therefore refitted the model excluding these variables, added interaction terms, and retained the only interaction term for which P<0.1 — the interaction between the number of cars available and difficulty walking (P=0.054) — although adding this interaction term made little difference to the estimated odds ratios for the other variables included in the model. In order to aid interpretation, we collapsed this pair of interacting variables into a single 2 x 2 composite variable and refitted the model (Table 5).

Addition of environmental correlates

In univariate analysis, nine 'environmental' variables met the criterion (P<0.25) to be considered in multivariate analysis: proximity to any major road; four individual items from the neighbourhood scale (attractiveness of surroundings, proximity to a park, proximity to shops, and road safety for cyclists); tertile of summary neighbourhood score; and three neighbourhood subscale scores derived from principal components analysis. We added these variables one at a time to the 'personal' multivariate model and identified four with P<0.1 which we considered worthy of further consideration for addition to the final model: proximity to any major road, proximity to shops, road safety for cyclists, and the subscale summary score for factor 2 ('low traffic'). However, the 'significant' result for proximity to any major road was confined to the comparison between one small category (401 to 500 metres) and the reference category (within 100 metres); there was no suggestion of a linear trend in the odds ratio with increasing distance. Furthermore, the P-values for the single neighbourhood scale items (proximity to shops, P=0.030; road safety for cyclists, P=0.023) were substantially smaller than that for the subscale summary score for factor 2 (P=0.086), and road safety for cyclists was one of the variables included in factor 2. We therefore fitted a final model containing age, housing tenure, distance to place of work or study, access to a bicycle, the composite variable reflecting the interaction between cars available and difficulty walking, day of travel diary, and two environmental variables: proximity to shops, and road safety for cyclists (Table 5).

Correlates of physical activity

Multivariate model of personal correlates

In univariate analysis, six 'personal' variables met the criterion (P<0.25) to be considered in multivariate analysis: age, housing tenure, working situation, distance to place of work or study, access to a bicycle, and BMI. When we entered these variables into a multivariate model along with sex, difficulty walking and the control variable for day of travel diary (study area of residence did not meet the criterion for inclusion), five variables (age, sex, working situation, distance to place of work or study, and access to a bicycle) no longer appeared to be significant. We therefore refitted the model excluding these variables and added interaction terms for each pairwise combination of all remaining variables, one at a time. We retained the only interaction term for which P<0.1 — the interaction between difficulty walking and body mass index (P=0.066), although adding this interaction term made little difference to the estimated odds ratios for the other variables included in the model. In order to aid interpretation, we collapsed this pair of interacting variables into a single 2 x 2 composite variable and refitted the model (Table 7).

Addition of environmental correlates

In univariate analysis, 13 'environmental' variables met the criterion (P<0.25) to be considered in multivariate analysis: proximity to existing motorway infrastructure; proximity to any major road; seven individual items from the neighbourhood scale (pleasantness for walking, proximity to a park, public transport, proximity to shops, routes for cycling, traffic volume, and safety crossing the road); tertile of summary neighbourhood score; and the three neighbourhood subscale scores derived from principal components analysis. We added these variables one at a time to the 'personal' multivariate model and identified three with P<0.1 which we considered worthy of further consideration for addition to the final model: proximity to existing motorway infrastructure, tertile of summary neighbourhood score, and traffic volume. However, the 'significant' results for proximity to existing motorway infrastructure and tertile of summary neighbourhood score were confined to the comparison between one category and the reference category; in neither case was there a suggestion of a linear trend in the odds ratio. We therefore fitted a final model containing housing tenure, the composite variable reflecting the interaction between body mass index and difficulty walking, and traffic volume, along with the control variable representing day of travel diary (Table 7).
